# The Use of Diode Low-Power Laser Therapy before In-Office Bleaching to Prevent Bleaching-Induced Tooth Sensitivity: A Clinical Double-Blind Randomized Study

**DOI:** 10.3390/dj11070176

**Published:** 2023-07-18

**Authors:** Felice Femiano, Rossella Femiano, Nicola Scotti, Ludovica Nucci, Antonino Lo Giudice, Vincenzo Grassia

**Affiliations:** 1 Multidisciplinary Department of Medical-Surgical and Dental Specialties, University of Study of Campania, “Luigi Vanvitelli”, 83138 Naples, Italy; 2 Department of Surgical Sciences, University of Turin, 10124 Torino, Italy; 3 Department of General Surgery and Surgical-Medical Specialties, University of Catania, Via S. Sofia 78, 95123 Catania, Italy

**Keywords:** in-office bleaching, tooth sensitivity, low-level laser therapy (LLLT), pain

## Abstract

Introduction: The present study aimed to investigate the effectiveness of low-level laser therapy (LLLT) use before in-office bleaching to prevent an increase in the risk and intensity of tooth sensitivity. Methods: Thirty patients were selected. Before bleaching with 38% hydrogen peroxide, the participants were randomly divided into two groups of 15 subjects. Test group: the patients’ teeth were subjected to a preliminary LLLT procedure by an 810 nm diode laser with 0.5 W for 30 s for an energy density of 15 J/cm^2^ and a group placebo. All patients were instructed to report their cold sensitivity experiences immediately, 1 h, 24 h, and 48 h after the end of bleaching via a VAS score. Results: The results obtained show an increase in VAS values for both groups (290 and 490 vs. 224 and 234 of baseline time of test and placebo group, respectively); afterward, the VAS value seemingly decreases at 1 h after the end of bleaching, approaching the baseline VAS for the test group (274) in comparison to the placebo group. Conclusions: The use of preliminary diode LLLT could represent a valid possibility to reduce the occurrence of tooth sensitivity post-whitening and shorten recovery time in cases where tooth sensitivity occurs.

## 1. Introduction

Nowadays, due to the increased aesthetic need of patients, tooth whitening is one of the most requested in-office practices, not only to eliminate intrinsic stains but also to improve the color of one’s teeth. Several approaches have been proposed to achieve adequate tooth bleaching, such as in-office bleaching, night-guard or home-applied bleaching, and over-the-counter systems. “Bleaching” or “teeth whitening” is the most conservative dental treatment for discolored teeth compared to other teeth whitening procedures such as resin-bonded composites, porcelain veneers, and crowns. Despite increasing interest in the home bleaching technique in recent years, in-office procedures are generally preferred by patients requesting fast results and hoping to avoid long wait times, as well as those who experience difficulty in wearing trays overnight due to concerns about ingesting whitening products at night [1,2]. The reactive oxygen molecules released by the whitening product are responsible for oxidizing dentin and enamel chromogens, yielding the desired effect. There are safety concerns correlated with the potential biological effects of free radicals, specifically free radicals of oxygen that are by-products or intermediates of hydrogen peroxide metabolism. A recent study reported that hydrogen peroxide (HP) and its derivatives could, without difficulty, pass enamel and dentine and access pulp tissue, initiating structural damage and inflammatory reactions [3]. 

Among the side effects reported in the literature, due to bleaching agents, cytotoxicity and deoxyribonucleic acid (DNA) damage are the most commonly described [4]. The least desired outcome by patients, at least in relation to in-office teeth whitening treatments with gel containing hydrogen peroxide (HP), is tooth sensitivity. Both the frequency and level of tooth sensitivity depend on the patient’s pain tolerance, the bleaching agent concentration, and, eventually, the heat emitted teeth whitening lamp used to accelerate the proceedings [5]. Studies have reported a prevalence rate of 65–85% for tooth sensitivity after in-office bleaching treatments using a high concentration of hydrogen peroxide alone or when the use of light-activation sources on bleaching agents is associated. The precise cause of teeth whitening-induced sensitivity is not yet known. However, the leading hypothesis has to do with the effects of hydrogen peroxide on one’s enamel and dentin. As the hydrogen peroxide product bleaches and removes the stains on one’s teeth, it also causes slight demineralization, which makes one’s teeth more porous and induces tooth sensitivity even though the dentinal tubules are not exposed. The other hypothesis is that the active molecules used for whitening can penetrate deeply into the tooth, inducing transient inflammation in the pulp along with an increase in interstitial fluid [6].

Generally, reports of pain and discomfort are mild, and the pain usually disappears quickly, but in some cases, the pain may be so severe and discomforting during treatment to the point where one pulls out from the decolorizing procedure [7]. Sometimes the symptoms can last for an indeterminate period of time after treatment, so much so that a fairly demanding desensitizing therapy is required [8].

Several studies have confirmed the success of using desensitizing agents in reducing tooth perceptivity after dulling [9,10]. The treatments proposed to reduce the symptoms of dental hypersensivity use agents containing potassium nitrate (to reduce the pain sensitivity by nerve depolarization) and fluoride or casein-phosphopeptide amorphous calcium phosphate (to increase to tooth mineralization) ahead, during, or after bleaching treatment. This common side effect can arise during or after the dental whitening; thus, deciding the type of treatment used to ameliorate the individual’s case and the planning of its duration can often produce stress for both the dentist and the patient [11]. 

Today, low-level laser therapy (LLLT) is increasingly used either in medicine or dentistry for its biostimulation, anti-inflammatory, and analgesic effects. These essential characteristics indicate that LLLT can decrease the harm and inflammation in pulp tissue induced by in-office bleaching procedures, therefore reducing the risk of increased tooth sensitivity from emerging. This procedure has been shown to be useful in several clinical and experimental studies [12,13,14]. The null hypothesis was that all the patients belonging to both the test and control groups reported symptoms referred to as dental hypersensitivity. As of today, there are no studies on the diode laser being used before in-office dental whitening procedures to counter bleaching-induced tooth sensitivity. In this study, we wanted to study the protective effect of using LLLT with a diode laser before an in-office teeth whitening procedure to avoid or reduce the risk and intensity of the onset of dental sensitivity.

## 2. Materials and Methods

This clinical double-blind randomized study consisted of a sample of 30 subjects who had explicitly requested teeth whitening procedures, necessitating the power of 80% to demonstrate an effect size of 30% with the Wilcoxon signed-rank test samples and with a first-type error of 0.05. The study was conducted in conjunction with a randomized double-blind and placebo-controlled clinical trial (Supplementary Material) and was registered in clinicaltrial.gov with the number: NCT05865275. The study was conducted in accordance with the ethical rules outlined by the Committee for human experimentation (institutional and national) and the Helsinki Declaration of 1975, as revised in 2013. Moreover, this study was approved with protocol number 1374/2019 by the Ethics Committee of AORN of Ospedale of Colli.

Only patients who were least 18 years old with teeth a shade darker than C2, according to the shade guide of Vita Classical A1-D4^®^- Germany, were recruited. 

All patients were in good general health with no dental caries, evident damage, or any restoration needed in their anterior teeth. Subjects who exhibited a negative vitality test for their teeth, from 1.4 to 2.4, were excluded from the study. Patients who used drugs such as analgesics, NSAIDs, or antioxidant products, or those with a smoking habit were excluded from the study, as well as patients with bruxism habits or with any pathologic defects such as gingival recession or dentin exposure. All patients included reported symptoms attributable to dental sensitivity. Two weeks before the bleaching procedure, the patients involved in the study were informed of professional and adequate oral hygiene upkeep measures and were told to brush their teeth twice per day with the same toothbrush and toothpaste during the research period. Every subject was informed, verbally and in writing, about the treatment procedures and was asked to sign an informed consent form before the study commenced. All the teeth from 1.4 to 2.4 of each patient that had to be subjected to whitening treatment were evaluated for dental sensitivity by employing the cool sensitivity test (baseline) [13].

Cold sensitivity Test (CST): To quantify tooth sensitivity, an air stimulus of 3 s with a temperature range of 19–20 °C at a distance of 2–3 mm from the test site was used. Before the investigation, each dental element was isolated with cotton rolls, while the neighboring teeth were in the gloved fingers of the dentist. After the procedure, the patients reported the level of sensitivity in each tooth in order to define the overall intensity of pain/discomfort they felt, using a visual analog scale (VAS) consisting of a 100 mm horizontal line on a sheet of paper with 0–10 points, where the ends ranged from 0 (the left side)—indicating no pain/discomfort—to 10 (the right side)—representing the worst possible pain they have ever experienced [14]. 

### 2.1. In-Office Bleaching Protocol

This RCT was designed in parallel with an allocation ratio of 1:1.

The thirty subjects selected were randomly divided into two groups (*n* = 15) using a Random Number Generator Software (Version N° 7.0 2023 Sobolsoft-Digital River GmbH- 50933 Cologne, Germany). Patient’s mean age was comparable in the two study groups (test group 23.4 ± 5.8 and placebo group 5.5 ± 6.2), and no significant difference in gender distribution was found between the two groups (8 males and 7 females, respectively, in the test and placebo group).

- Test group: Before bleaching, the patient’s teeth were subjected to an irradiation procedure via LLLT using a diode laser (Soft Touch; 810 nm, 5 W, Creation Medical Laser-Ennebi Elettronica S.r.l. Novedrate—Como- Italy). The laser probe (a fiber of 400 µm diameter) was positioned in contact mode, with the enamel of the tooth being irradiated for 30 s in continuous-wave using 0.5 W and with the operator employing a horizontal and vertical motion to cover the whole area. Each tooth was subjected to an energy density of 15 J/cm^2^. Subsequently, each patient underwent the teeth whitening procedure using the protocol recommended by the product’s manufacturer. A light-cured resin dam (OpalDam Kit, Ultradent; Corsico, MI, Italy) was used to isolate the gingiva from the bleaching gel. A 38% ready-to-use hydrogen peroxide-containing gel (The Smile^®^ Strong; Wintek-Monopoli-Bari-Italy) was applied from the first premolar on the right to the first premolar on the left of both sides of the jaw for a total period of 20 min for a 1 single whitening session [3,4].
- Placebo group: Before bleaching, to avoid psychological interference with respect to the outcomes of the study, all subjects in the group were subjected to a preventive laser procedure applying the identical parameters used for the group test, except the device was kept in idle mode. Otherwise, the teeth whitening procedure was identical to that of the test group.

### 2.2. Following the Bleaching Treatment, the Gel Was Rinsed Off

The laser procedure and the whitening procedure were performed by two different clinical operators who were not aware of the study groups each subject belonged to.

Subsequently, the first operator, who was employed to collect VAS scores at baseline and was unaware of the assigned clinical protocols, again subjected all of the study’s participants to CST, asking them to record the degree of dental sensitivity for each bleached tooth, instructing the subjects to record their level of pain immediately after the procedure and 1 h, 24 h, and 48 h after the end of bleaching treatment using the VAS score. 

### 2.3. Statistical Analysis

To quantify the cold discomfort experience by each patient, a single VAS value was considered, which was obtained from the sum of the individual VAS values from each tooth exposed to the cold test, while for the comparison of discomfort between the two groups, the total VAS score of each group was used, and this was derived from the sum of the VAS values of each patient collected at the different times after the procedure (immediately after the procedure and 1 h, 24 h, and 48). The VAS values of each group were compared with each other and also compared with the baseline/reference values for the different times after the procedure. The means and standard deviations were calculated, and ANOVA was used to compare the measurements in each group. A paired *t*-test was used to assess discomfort before and after the procedure. Statistical analysis was performed using GraphPad Prism software version 9 (by Dotmatics, San Diego CA, USA.) The significance level for all tests was set at *p* < 0.05.

## 3. Results

All 30 participants completed the study. 

**Figure 1:**
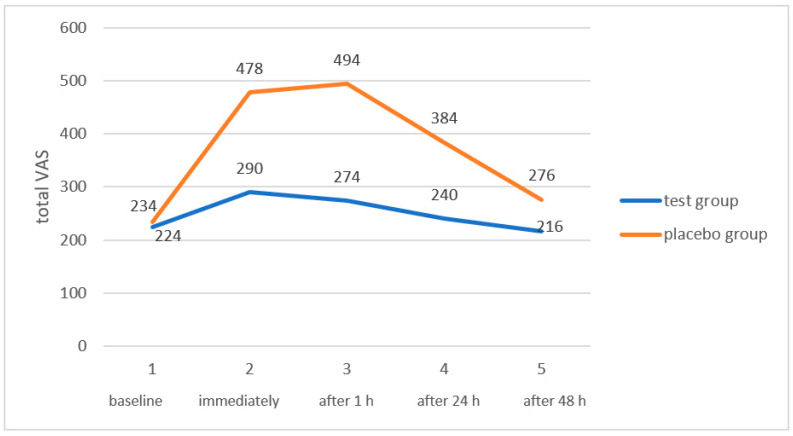
Comparison of total VAS in both study groups during the observation period.

Table 1 shows the total VAS values (mean and standard deviation, SD) for each study group at the different time points of the clinical trial. The comparison of the total VAS scores for each group study within the same study time revealed statistically significant values after using the Wilcoxon signed-rank test for times of immediately after the procedure, 1h after, and 24 h after, while there was no significant difference for the VAS values 48 h after the whitening procedure (Table 1 and Figure 1). The comparison of the VAS score of each group with the corresponding value at baseline shows high significance for both groups immediately after the procedure and only with the equivalent VAS value of the placebo group after 1 h and 24 h after whitening.

## 4. Discussion

Many researchers are trying to find increasingly effective remedies to counteract the symptoms of bleaching-induced tooth sensitivity, using different substances that induce remineralization or reduce sensitivity with quite satisfactory but sometimes not immediate results. Less time has been devoted to the study of substances for therapeutic procedures designed to be used before tooth whitening to try to preemptively reduce the risk and intensity of the onset of tooth sensitivity after in-office bleaching; however, thus far, studies that have considered this have reported disappointing results [6,7,8,9,10].

Recently, Vochikovski L et al. (2022) [15], in a split-mouth, double-blind, placebo-controlled study consisting of 50 participants, proved that a desensitizing gel composed of 10% calcium gluconate, 0.1% dexamethasone acetate, 10% potassium nitrate, and 5% glutaraldehyde applied on the teeth for 10 min before in-office bleaching does not reduce the risk and the intensity of tooth sensitivity. Ortega-Moncayo MG et al. (2021) [16] evaluated the effectiveness of applying a dentifrice containing 5% potassium nitrate before and during in-office dental bleaching in reducing bleaching-induced tooth sensitivity using a test and control group. 

The results obtained showed no significant difference in the absolute risk and intensity of tooth sensitivity between the test and control groups. Gümüştaş B et al. (2022) [17], compared the local use of different remineralization agents (casein phosphopeptide-amorphous calcium phosphate, neutral sodium fluoride gel, or nano-hydroxyapatite solution) before in-office bleaching to decrease bleaching induced sensitivity. The results of the study showed that the preliminary use of remineralization agents before in-office bleaching did not reduce tooth sensitivity. There are several scientific studies on the use of lasers in dental whitening with the aim of improving or aiding the whitening effect of the products used, whereas the use of lasers to prevent bleaching-induced tooth sensitivity has been scarcely reported [18,19]. 

Our study valued the preventive effect of the low power of a diode laser on the occurrence of tooth sensitivity after in-office bleaching. Tooth sensitivity is a common sense of discomfort that is considered an undesirable effect of in-office dental whitening treatment in the absence of exposure of the dentin. Its cause can be related to aggressive treatment or the prolonged use of enamel-bleaching substances with excessive pH lowering capabilities. This causes an excessive widening of interprismatic spaces which facilitates the progression of oxidizing agents toward the dental pulp with inflammation and fluid extravasation pushing towards the dentinal tubule. The movement of intratubular fluid, with modify of pressure parameters is the basis of Brännström’s hydrodynamic theory [20,21]. The symptomatology is characterized by a sense of increasing pain after contact with foods or liquids with extreme thermal variations or hyperosmolar liquids. Tooth sensitivity experienced after whitening is often considered a transient discomfort, usually spontaneously disappearing within 24/48 h after enamel remineralization and a reduction in interprismatic spaces takes place. In our study, most pain sensitivity complaints were reported within the first 24 h after bleaching, and these data are in agreement with the results of other authors [22,23,24,25,26].

Sometimes and especially in patients with a lower pain threshold, symptoms can be more pervasive and long-lasting. This can be a source of anxiety both for the clinician and the patient. There are different remedies that can be used to try to counteract dental hypersensitivity either using remineralizing agents (sodium fluoride, casein phosphopeptide, arginine, and calcium carbonate), with occlusion or narrowing of dentinal tubules, and using depolarizing agents (nitrate potassium). effective on nerve conduction with block the exposed dentine tubules Many clinical studies have demonstrated the efficacy of low-power diode lasers alone or in concert with fluoride gel applied to the enamel surface in reducing or removing pain from dental hypersensitivity after whitening [27,28].

Our study investigated the protective effect of a low-power diode laser on the risk and intensity of bleaching-induced tooth hypersensitivity.

The study data indicate that, in both groups, there was an increase in dental sensitivity immediately after the whitening treatment, even if this was lower in intensity for patients in the test group (VAS 290 vs. 224 at baseline) compared to the placebo group (VAS 478 vs. 234 at baseline). These values tended to decrease after 1 h for the test group (274 vs. 494 of the placebo group) and after 24 h (240 vs. 384 of the placebo group) before reaching the baseline values after 48 h in both groups, with VAS values of 216 and 260 for the test and placebo groups, respectively. The authors hypothesize that the lower pain felt for dental sensitivity, as evidenced by the VAS scores in the test group, is due to the effects of the biostimulation of the diode laser used at low power in conjunction with the stimulation of odontoblasts, which leads to the formation of new dentin, and the anti-inflammatory effects on the dental pulp; however, the analgesic effect of the diode during LLLT on the pulp tissue due to the depolarizing effect on C and Aδ nerve fibers plays a key role [29].

The direct action on nerve transmission could explain the lower pain from dental hypersensitivity recorded for patients in the test group recorded immediately after the teeth whitening procedure, while the faster resolution of symptoms after 24 h compared to patients in the placebo group could be related to the anti-inflammatory effect of the laser and the production of irregular secondary dentin.

The VAS is a commonly known method used to assess responses to discomfort, and it has many advantages over a descriptive rating scale. Through VAS, a subject can easily express their response to a changing pattern of pain/discomfort, meaning that it is of value when assessing discomfort in subjects. Cross-group comparisons of responses should be performed with care. Congruent results for different people at the same time, or even by the same subject at different times, do not permit us to make statements about the quality of the perceptions of discomfort; identical scores may have different implications. The unmarked scale we used lacked a neutral point, so a subject did not know where the range changed from ‘mild pain’ to ‘mild no pain’. Scores placed centrally on the scale could beb attributed to uncertainty, distractions, or even a lack of interest among patients. Most individuals reported discomfort to some degree, but sometimes, it had a very small impact on their daily life. These results agree with previous studies on discomfort levels following bleaching procedures [23,24]. Where necessary, we recommend that analgesics are prescribed after the procedure, and patients are advised to change their diet to soft food in the first 2 to 3 days after bleaching. Good oral hygiene is important to avoid localized periodontitis. Therefore, the standard of one’s oral hygiene is not a criterion the clinician should consider when deciding on the type of protocol to use.

A limitation of this study is that the results presenter here are inextricably linked to the use of a single scanner and therefore the results that have emerged from the study refer to the potential of that device alone. The same problem can be related to using a product with a certain percentage of peroxide. As much as it is frequently noted in the literature, it is important to state that different percentages could lead to different results. The authors intend to expand the sample size and compare other work protocols in the hopes that it will allow them to better define which procedures can minimize discomfort after bleaching.

## 5. Conclusions

In light of the results obtained, the use of a low-power laser procedure for 30 s on each tooth to induce dental whitening can reduce the appearance of dental hypersensitivity symptoms after teeth whitening.

## Figures and Tables

**Table 1:** The total, means, and standard deviations (SD) of VAS scores in each study group at different time points.

Groups	Baseline (b)	Immediately (t1)		After 1 h (t2)		After 24 h (t3)		After 48 h (t4)	
**Test (T)**	224 (14.9; 10.4)	290 (19.3; 9.1)		274 (18.3; 8.2)		240 (14.4; 7.5)		216 (16; 10.9)	
**Placebo (C)**	234 (15.6; 10.1)	478 (31.9; 10.8)		494 (32.9; 9.9)		384 (25.6; 7.5)		260 (17.3; 6.4)	
	*p*								
**T vs. C**	0.3606	0.0020		0.0028		0.0054		0.809	
		T	C	T	C	T	C	T	C
**b vs. t1,t2,t3,t4**		0.0039	0.0001	0.6221	0.0002	0.5	0.0111	>0.999	0.498

## Data Availability

All data related to this research article are available from the corresponding author, Felice Femiano.

## Supplementary Materials

The following supporting information can be downloaded at: https://www.mdpi.com/article/10.3390/dj11070176/s1.

## Abbreviations

LLLT	low-level laser therapy
HP	hydrogen peroxide
DNA	deoxyribonucleic acid
CST	Cold sensitivity Test
VAS	visual analog scale
SD	standard deviations

## References

[1] Parlar Oz O., Demirkol N. (2021). Effectiveness of in-office bleaching treatment with different activation techniques on tooth color changes and sensitivity: A randomized clinical trial. Am. J. Dent..

[2] Ferraz N.K.L., Nogueira L.C., Neiva I.M., Ferreira R.C., Moreira A.N., Magalhães C.S. (2019). Longevity, effectiveness, safety, and impact on quality of life of low-concentration hydrogen peroxides in-office bleaching: A randomized clinical trial. Clin. Oral. Investig..

[3] Ahrari F., Akbari M., Mohammadpour S., Forghani M. (2015). The efficacy of laser-assisted in-office bleaching and home bleaching on sound and demineralized enamel. Laser Ther..

[4] Patri G., Agnihotri Y., Rao S.R., Lakshmi N., Das S. (2013). An in vitro spectrophotometric analysis of the penetration of bleaching agent into the pulp chamber of intact and restored teeth. J. Clin. Diagn. Res..

[5] Vochikovski L., Favoreto M.W., Rezende M., Terra R.M.O., Gumy F.N., Loguercio A.D., Reis A. (2022). Use of infrared photobiomodulation with low-level laser therapy for reduction of bleaching induced tooth sensitivity after in-office bleaching: A double-blind, randomized controlled trial. Lasers Med. Sci..

[6] Irusa K., Abd Alraheam I., Nguyen Ngoc C., Donovan T. (2022). Tooth whitening procedures: A narrative review. Dent. Rev..

[7] Carregosa Santana M.L., Leal P.C., Reis A., Faria-E-Silva A.L. (2019). Effect of anti-inflammatory and analgesic drugs for the prevention of bleaching-induced tooth sensitivity: A systematic review and meta-analysis. J. Am. Dent. Assoc..

[8] Maran B.M., Vochikovski L., Hortkoff D.R.D.A., Stanislawczuk R., Loguercio A.D., Reis A. (2020). Bleaching sensitivity with a desensitizing in-office bleaching gel: A randomized double-blind clinical trial. Quintessence Int..

[9] Godoy C.E., Consani S., Guimarães A.T., Laurindo B.M., Mendonça M.J., Camilotti V. (2021). Effect of two desensitizing agents applied previous to in-office bleaching on the degree of whitening and dentin sensitivity: A randomized, controlled, double-blind clinical trial. Am. J. Dent..

[10] Llena C., Esteve I., Rodríguez-Lozano F.J., Forner L. (2019). The application of casein phosphopeptide and amorphous calcium phosphate with fluoride (CPP-ACPF) for restoring mineral loss after dental bleaching with hydrogen or carbamide peroxide: An in vitro study. Ann. Anat. Anat. Anz..

[11] Pierote J.J.A., Barbosa I.F., Prieto L.T., Lima D.A.N.L., Paulillo L.A.M.S., Aguiar F.H.B. (2019). Effects of desensitizing dentifrices on the reduction of pain sensitivity caused by in-office dental whitening: A double-blind controlled clinical study. Clin. Cosmet. Investig. Dent..

[12] Naghsh N., Kachuie M., Bijari M., Birang R. (2022). Evaluation of the effects of 980 and 810-nm high-level diode lasers in treating dentin hypersensitivity: A double-blinded randomized clinical trial. Dent. Res. J..

[13] Femiano F., Femiano L., Nucci L., Grassia V., Scotti N., Femiano R. (2022). Evaluation of the Effectiveness on Dentin Hypersensitivity of Sodium Fluoride and a New Desensitizing Agent, Used Alone or in Combination with a Diode Laser: A Clinical Study. Appl. Sci..

[14] Femiano F., Femiano R., Femiano L., Aresu G., Festa V.M., Rullo R., Perillo L. (2018). Effectiveness of low-level diode laser therapy on pain during cavity preparation on permanent teeth. Am. J. Dent..

[15] Vochikovski L., Favoreto M.W., Rezende M., Terra R.M.O., da Silva K.L., Farago P.V., Loguercio A.D., Reis A. (2023). Effect of an experimental desensitizing gel on bleaching-induced tooth sensitivity after in-office bleaching-a double-blind, randomized controlled trial. Clin. Oral. Investig..

[16] Ortega-Moncayo M.G., Aliaga-Sancho P., Pulido C., Gutierrez M.F., Rodriguez-Salazar E., Burey A., León K., Román-Oñate Y., Galvao Arrais C.A., Loguercio A.D. (2022). Is the use of a potassium nitrate dentifrice effective in reducing tooth sensitivity related to in-office bleaching? A randomized triple-blind clinical trial. J. Esthet. Restor. Dent..

[17] Gümüştaş B., Dikmen B. (2022). Effectiveness of remineralization agents on the prevention of dental bleaching induced sensitivity: A randomized clinical trial. Int. J. Dent. Hyg..

[18] Méndez Romero J.M., Villasanti Torales U.A., Villalba Martínez C.J. (2020). Efficacy of laser application in dental bleaching: A randomized clinical controlled trial. Am. J. Dent..

[19] Brugnera A.P., Nammour S., Rodrigues J.A., Mayer-Santos E., de Freitas P.M., Brugnera Junior A., Zanin F. (2020). Clinical Evaluation of In-Office Dental Bleaching Using a Violet Light-Emitted Diode. Photobiomodulation Photomed. Laser Surg..

[20] Kwon S.R., Pallavi F., Shi Y., Oyoyo U., Mohraz A., Li Y. (2018). Effect of Bleaching Gel Viscosity on Tooth Whitening Efficacy and Pulp Chamber Penetration: An In Vitro Study. Oper. Dent..

[21] Brännström M., Aström A. (1972). The hydrodynamics of the dentine; its possible relationship to dentinal pain. Int. Dent. J..

[22] Kossatz S., Dalanhol A.P., Cunha T., Loguercio A., Reis A. (2011). Effect of light activation on tooth sensitivity after in-office bleaching. Oper. Dent..

[23] Paula E., Kossatz S., Fernandes D., Loguercio A., Reis A. (2013). The effect of perioperative ibuprofen use on tooth sensitivity caused by inoffice bleaching. Oper. Dent..

[24] Faria-E-Silva A.L., Nahsan F.P.S., Fernandes M.T.G., Martins-Filho P.R.S. (2015). Effect of preventive use of nonsteroidal anti-inflammatory drugs on sensitivity after dental bleaching: A systematic review and meta-analysis. J. Am. Dent. Assoc..

[25] De Paula E.A., Kossatz S., Fernandes D., Loguercio A.D., Reis A. (2014). Administration of ascorbic acid to prevent bleaching induced tooth sensitivity: A randomized triple-blind clinical trial. Oper. Dent..

[26] Carneiro A.M.P., Barros A.P.O., de Oliveira R.P., de Paula B.L.F., Silva A.M., de Melo Alencar C., Silva C.M. (2022). The effect of photobiomodulation using low-level laser therapy on tooth sensitivity after dental bleaching: A systematic review. Lasers Med. Sci..

[27] Yahya G., AlAlwi A., Shurayji F., Baroom W., Rajeh M., AbdelAleem N. (2022). Effectiveness of sodium fluoride varnish and/or diode laser in decreasing post-bleaching hypersensitivity: A comparative study. Saudi. Dent. J..

[28] Coleton S. (1998). Sensitivity and laser treatment. J. Am. Dent. Assoc..

[29] Jena A., Shashirekha G. (2015). Comparison of efficacy of three different desensitizing agents for in-office relief of dentin hypersensitivity: A 4 weeks clinical study. J. Conserv. Dent..

